# Initial trail selection in mantrailing dogs under double-blind field conditions: a statistical evaluation of scent discrimination performance

**DOI:** 10.1016/j.fsisyn.2026.100682

**Published:** 2026-05-04

**Authors:** Vera Volmary

**Affiliations:** Faculty of Environment and Natural Sciences, Brandenburg University of Technology Cottbus–Senftenberg, Cottbus, Germany

**Keywords:** Mantrailing, Scent discrimination, Initial trail choice, Condition bias, Field validation, Reproducibility

## Abstract

This study examined, under environmentally realistic yet strictly controlled field conditions, whether mantrailing dogs can reliably discriminate the individual human scent of a target person at the point of initial trail selection. A total of 70 operationally deployed dogs with heterogeneous training backgrounds participated in a randomised, double-blind field study comprising single-trail and scent-discrimination scenarios under standardised start conditions. Both positive and negative scent setups were included.

Across all test conditions, directional decision success rates were consistent with chance expectation. All 95% confidence intervals fully overlapped with the chance range predicted by the corresponding binomial model for the respective task. Neither training level, organisational background, breed, age, nor recorded environmental parameters showed a measurable influence on the probability of correct trail selection. Repeated testing did not lead to performance stabilisation; success rates remained within chance expectations across all test rounds.

Qualitative behavioural observations indicated frequent reliance on non-olfactory cues, including socially or contextually influenced search decisions, consistent with the presence of condition bias. Teams that appeared successful in individual trials did not demonstrate reproducible discrimination performance above chance upon retesting.

Under controlled field conditions, mantrailing dogs did not show a reproducible above-chance ability to discriminate individual human scents during initial trail selection. These findings indicate that observed training or operational successes cannot be assumed to reflect a validated individual-scent discrimination capability and underline the need for rigorously controlled, reproducible study designs when evaluating mantrailing performance in applied contexts.

## 1 Introduction

The forensic use of mantrailing dogs is widespread in police practice and has repeatedly been subject to judicial scrutiny, particularly with regard to evidential value and admissibility in court proceedings [[1], [2], [3]]. Despite this high operational relevance, empirical validation of the performance of mantrailing dogs under realistic field conditions remains limited. While training and operational standards provide normative frameworks for this domain [4,5], and methodologically controlled studies have demonstrated dogs’ fundamental ability to discriminate between individual human scents [[6], [7], [8]], there is still a lack of studies that systematically analyse initial trail selection between competing scent trails under controlled, blinded, and statistically evaluable conditions.

A substantial proportion of the empirically established literature on scent discrimination is based on laboratory-based, highly controlled experimental designs, particularly scent identification line-ups and match-to-sample paradigms [[6], [7], [8]]. These studies demonstrate that dogs are capable of discriminating between human odours under controlled conditions; however, transferability of these results to operational mantrailing scenarios is limited. Field-based studies, while offering greater environmental validity, frequently fail to achieve comparable control over potential social, contextual, or expectation-based cues. As a result, it often remains unclear to what extent observed search decisions are driven exclusively by olfactory information [9,10]. Consequently, several of these studies have been controversially discussed in the scientific literature with respect to their methodology and interpretative validity [9,[11], [12], [13]].

From a forensic perspective, the initial decision point is of particular importance, as it determines which trail is subsequently followed and must therefore be analytically distinguished from downstream parameters such as endurance or trail persistence [2,3]. Methodological analyses and experimental studies indicate that robust conclusions regarding the evidential value of mantrailing results are difficult to draw when investigations lack adequate blinding, randomisation, or appropriate control conditions, and when non-olfactory influences such as expectation effects and cueing are insufficiently controlled [4,5,[11], [12], [13], [14], [15], [16], [17], [18], [19]].

In addition to experimentally demonstrated bias effects, practice-oriented literature on human–dog interaction describes that handlers, in everyday working contexts, may frequently and unintentionally provide cues through body posture, movement patterns, or habitual decision routines that can influence canine search behaviour [[14], [15], [16], [17],^20^,^21^]. Learning-theoretical models further indicate that stimulus predictability and pre-existing expectations can systematically affect attention allocation and the weighting of new information [22,23]. Together, these findings underscore the necessity of consistently controlling and empirically testing expectation- and context-related factors, even under field-realistic conditions.

Against this background, empirical evidence on initial trail selection under field-realistic, randomised, and double-blind conditions remains scarce. The central research gap therefore does not concern whether dogs possess olfactory discrimination capabilities per se, but whether mantrailing dogs can reliably select the correct individual trail at the decision point under realistic operational conditions and controlled expectations [[1], [2], [3],^19^]. Of particular importance is the systematic inclusion of negative scent setups, as only these allow for the assessment of specificity, false-positive decisions, and individual decision thresholds—key prerequisites for the forensic interpretation of search results [[1], [2], [3], [4], [5]].

The present study addresses this research gap by investigating operational mantrailing teams under controlled yet field-realistic conditions. Discrimination performance at the level of initial trail selection was statistically evaluated under strict control of non-olfactory influences.

Contemporary research on detection and scent-working dogs conceptualises search performance not as a static, innate ability but as the outcome of learning- and experience-dependent decision-making processes. Training influences not only sensitivity and specificity in detection tasks but also the processing of olfactory information, generalisation performance, and the development of search and decision strategies, including the integration of non-olfactory cues such as social, contextual, or handler-related information [[15], [19], [24]]. Extensive and systematic training over prolonged periods is therefore considered an important prerequisite for achieving stable and reproducible performance in complex scent tasks, including matching-to-sample paradigms and human scent discrimination [[6], [13], [17]].

In practical training and operational contexts, however, contextual and social cues are often only partially controllable, making it difficult to determine to what extent training experience genuinely contributes to valid, person-specific trail selection. Whether reproducible individual scent discrimination at the level of initial trail selection can be demonstrated under field-realistic, yet randomised and double-blind conditions with extensive control of non-olfactory influences has not yet been systematically investigated.

The null hypothesis of the present study was that, under randomised, double-blind field conditions, mantrailing dogs select the trail associated with a presented scent article at a rate not exceeding chance expectation.

This includes both correct initial trail selections when a target trail was present (true positives) and correct decisions not to initiate a trail when no target trail was present (true negatives).

The alternative hypothesis was that mantrailing dogs would select the correct trail, or correctly reject a trail, at a rate significantly exceeding chance expectation under the same conditions.

## 2 Materials and methods

### 2.1 Study design

The study was designed as a prospective, randomised, double-blind field experiment to assess whether mantrailing dogs can discriminate individual human scents at the level of initial trail selection under operationally realistic yet controlled conditions. Both single-trail and scent-discrimination scenarios were conducted.

The primary endpoint was the dog's initial directional decision at the starting point, operationalised as the dominant core direction that emerged after search initiation.

Initial directional decisions were evaluated relative to the geometric orientation of the target trail, which defined the target vector. Where the target runner departed, for example, in a northward direction, stable orientation along this vector was considered directionally consistent.

Deviations from the target vector were acceptable insofar as clear spatial attribution to the target trail remained possible. If a dog showed stable and sustained drift markedly toward eastward or westward directions, no unambiguous orientation toward the target trail geometry could be inferred, as the search trajectory approached competing trails in close spatial proximity. Such trajectories were therefore not classified as directionally consistent with the target trail.

Brief investigation of alternative directions and temporary interruptions were not considered errors, provided that subsequent behaviour indicated a consistent spatial orientation towards the selected trail. In cases of ambiguous search behaviour, handler feedback documented immediately after the search was used solely to support classification as inconclusive and did not influence the primary endpoint.

To minimise expectancy effects and non-olfactory influence, a multi-layered randomisation and blinding protocol was implemented. Allocation of teams to test intersections followed constrained randomisation accounting for logistical factors. Selection of scent containers (identical glass jars) containing runner scent articles or a negative control article was fully randomised by draw. Runner directions at each start point were likewise assigned by draw. The target scent article was selected by the handler from a set of five visually identical containers (four runners, one negative control). This procedure ensured double-blinding with respect to both target person and corresponding trail.

**Fig. 1:**
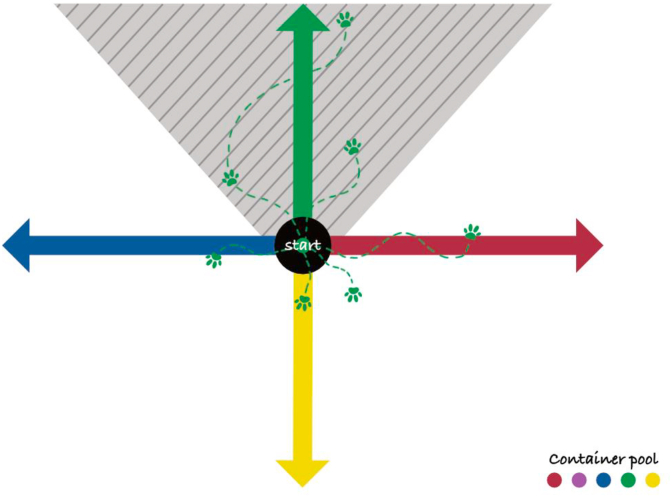
Schematic representation of the initial trail choice at a four-way intersection. Coloured arrows indicate the four scent trails laid by the runners. The grey shaded area represents the core direction sector used to operationalise the initial directional decision (here exemplarily shown for the green scent trail). Exploratory movements outside the core sector were permitted. The container pool represents the randomised selection of one scent container from a set of five identical containers, comprising four target scent containers and one negative container (colour assignment varied between trials).

A schematic representation of the initial trail choice at a four-way intersection and the operationalisation of the initial directional decision is shown in Fig. 1.

The aim of the study was not to compare individual team performance but to assess whether fundamentally trained mantrailing dogs, considered as a group, demonstrate reliable individual scent discrimination under field conditions.

### 2.2 Study participants

A total of 70 dog–handler teams participated in the study. The sample comprised equal numbers of female and male dogs (35 each); 59% of the dogs were neutered. Dogs ranged in age from 2 to 13 years (mean ± SD = 6.26 ± 2.83 years), representing a broad age distribution.

Overall, 49 dogs (70%) were classified as purebred and 21 dogs (30%) as mixed breed, representing 35 different breeds with no single breed being overrepresented. The sample was predominantly characterised by hunting-type breed traits in both purebred dogs and the majority of mixed-breed dogs; herding and protection breeds were considerably less represented. A complete listing of breed affiliation for each dog is provided in Supplementary Table S1.

Mantrailing experience ranged from less than one year to more than eight years. Dogs were trained in heterogeneous organisational contexts, including operational, sport, and recreational settings, with corresponding variation in examination and certification status. At the time of testing, approximately 60% of the dogs had passed a formal mantrailing examination or partial examination, while the remaining teams had not yet completed any formal certification.

Individual-level data on dog characteristics and training background are provided in Supplementary Table S1 and S2.

### 2.3 Test locations and environmental control

Testing was conducted at two decommissioned military facilities offering large, structurally diverse areas with varying substrates (asphalt, gravel, grass), buildings, open spaces, and vegetation. One site was regularly used for training; the other was unused, minimising third-party contamination.

Start points and trail areas were kept free of human activity for at least 18–24 h prior to testing. Trails were laid and worked throughout the day from early morning to late afternoon or evening. Adjacent start points were not used consecutively. Environmental parameters (temperature, relative humidity, precipitation, cloud cover) were recorded for each search.

### 2.4 Trail planning and standardisation

Each start point consisted of a four-arm intersection. Four trails were pre-planned per intersection, extending in different directions over comparable substrates.

Trail length varied between testing rounds but followed a structured protocol. In the first testing round, trail lengths ranged from approximately 200 m to 470 m. In repeated testing rounds, trails were systematically extended (approximately 490 m to 660 m), solely to increase spatial separation of trail endpoints and thereby improve the unambiguous attribution of the initial core running direction to the target vector.

Trail age at search initiation was standardised across all trials. Dogs typically began searching approximately 15 min after trail laying. In some cases, organisational factors resulted in slightly delayed starts; however, trail age consistently remained within a predefined window of approximately 15 to 30 min. Start times were scheduled according to this protocol, ensuring comparable scent-age conditions irrespective of trail length or testing round.

Trail length was considered secondary, as the analytical focus was on initial directional decision. At the end of each trail, runners entered enclosed buildings to exclude high-air-scent localisation (high scenting). Dogs therefore had to base their initial decision on the scent article and ground-level scent information.

All trails were planned in advance, recorded using GPS, photographically documented, and provided to runners as standardised trail schematics, ensuring a high degree of procedural consistency and reproducibility across trials.

### 2.5 Scent articles

Plain black socks were used as scent articles due to their practical relevance, high scent intensity, and visual indistinguishability. Socks were worn by the runners during normal daily activities for approximately one day, without any additional standardisation of wear duration, to reflect realistic operational conditions. Each scent article was subsequently stored individually in identical, colour-coded glass containers with metal lids.

Glass was chosen due to minimal adsorption of volatile organic compounds. Containers were standardised cleaned and thermally conditioned between uses. All scent articles were handled according to a fixed protocol to maintain blinding and minimise contamination.

### 2.6 Test procedures

Each team completed two test formats:

***Task 1: Single-trail test:*** At a given intersection, one person chose one of four pre-planned trail descriptions and laid the corresponding trail accordingly. The dog's task was to identify the only existing trail and follow it in the initial direction. The runner was known or unknown to the dog depending on availability. Trail direction was randomised by draw. The test was double-blind.

***Task 2: Scent-discrimination test:*** Four unfamiliar persons simultaneously laid trails from the same starting point in different directions (Fig. 1). All four provided scent articles; an additional scent article from a non-present person served as a negative control. The dog was required to select the correct individual trail based on the presented scent article chosen by the handler by draw.

Handlers were free to use any search strategy, including perimeter searches. Occurrence of perimeter behaviour was documented but not restricted.

### 2.7 Randomisation and blinding

Randomisation was implemented at multiple levels:

• allocation of trail directions,
• selection of target person,
• inclusion of negative setups (in Task 2).

For each start point, multiple pre-planned trail schematics were available and known exclusively to the study coordinator. Random allocation of trails was a central prerequisite to ensure strict double-blinding, as the study coordinator accompanied the search and could otherwise have conveyed—albeit unintentionally—implicit cues regarding trail identity or target status.

Handlers and accompanying personnel were unaware of trail direction, target person, and the presence of positive or negative setups, ensuring effective double-blind conditions throughout the search procedure.

### 2.8 Outcome classification

Search outcomes were classified using established diagnostic categories:

• True Positive (TP)
• False Positive (FP)
• False Negative (FN)
• True Negative (TN)

A result was considered correct if the dog selected the correct initial trail direction. Strict trail fidelity was not required. Ambiguous behaviour with repeated directional changes was classified as inconclusive.

### 2.9 Retesting procedure and selection criteria

Retesting was conducted to assess the reproducibility of initial trail selection performance rather than to increase statistical power, as repeated trials were neither independent nor intended for pooled statistical analysis.

Selection for retesting was based on outcome categories derived from the scent-discrimination task. Teams were invited to subsequent test rounds if they had achieved either (i) a correct outcome (true positive or true negative) or (ii) an inconclusive result with partial behavioural indications of potential target-oriented processing (e.g. intermittent increased interest in the correct trail sector or lack of clear differentiation in negative setups), without meeting the criteria for a correct outcome.

Correct outcomes (true positives and true negatives) were defined according to the criteria described above, based on the initial directional decision within the predefined target sector (Fig. 1). True negatives were assigned only when no trail was adopted in a sustained manner and no preferential following relative to alternative trails was observed.

Retesting was conducted iteratively. Following the first test round (n = 70 teams), 20 teams meeting the above criteria were invited to a second round. Based on the same selection criteria, 5 teams were subsequently invited to a third round.

Retests were conducted on separate days, typically on subsequent weekends, and never on the same day as the initial test. The decision to implement retesting was made during the course of data collection, once it became apparent that overall performance remained within chance expectation. Invitations to retesting were issued after outcome classification based on documented search trajectories.

The two test formats (single-trail and scent-discrimination tasks) were generally conducted on separate days to minimise potential fatigue effects. In a minority of cases (10 teams), both tasks were conducted on the same day due to logistical constraints; in these instances, tests were separated by several hours.

### 2.10 Statistical analysis

The primary endpoint was the proportion of correct initial directional decisions in scent-discrimination tests. Success rates were reported as point estimates with 95% confidence intervals (Wilson method). Exact binomial tests were used to assess deviation from chance expectation (p_0_ = 0.20 in Task 2 or 0.25 in Task 1).

Individual dogs participated in one to three scent-discrimination trials, depending on predefined selection criteria for repeated testing. Participation in subsequent rounds was determined solely by performance characteristics in the preceding round and not by availability.

Repeated trials were not pooled at the individual level. Each testing round was analysed separately and in its entirety against the corresponding chance expectation. In addition, an aggregated analysis across all trials was conducted independently. Repeated testing served exclusively to assess reproducibility and not to increase statistical power; therefore, no mixed-effects or repeated-measures models were applied.

Exploratory analyses assessed associations with training and environmental variables. As no systematic deviations from chance were observed, no multivariable regression models were applied. All analyses were conducted using an R-based statistical environment.

## 3 Ethics statement

The study was conducted in accordance with applicable ethical and legal standards and involved non-invasive behavioural field testing of already trained mantrailing dogs under routine training conditions, using standardised procedures. Participation was voluntary, informed consent was obtained from all handlers, and animal welfare was continuously monitored. In accordance with applicable regulations, no approval by an institutional animal ethics committee was required.

## 4 Results

A total of 166 field trials were conducted with 70 dog–handler teams, comprising 72 single-trail tests and 94 scent-discrimination trials, performed under realistic and variable environmental conditions.

### 4.1 Single-trail performance

Dogs showed correct orientation toward the target trail in 28% of single-trail tests, with a correct find rate of 15%. In the remaining 72% of trials, no sufficient orientation toward the target trail was observed, consistent with chance-level performance.

### 4.2 Scent discrimination

Across all 94 scent-discrimination trials, 18% of decisions were correct (true positives and true negatives), while 82% were incorrect, consistent with chance-level performance. When a target trail was present, correct discrimination occurred in 19.7% of trials; correct rejection in negative trials occurred in 11.1%.

The correct target person was located in 9% of discrimination trials, whereas an incorrect person was found in 19%, with frequent near-approaches to incorrect hiding locations.

**Fig. 2:**
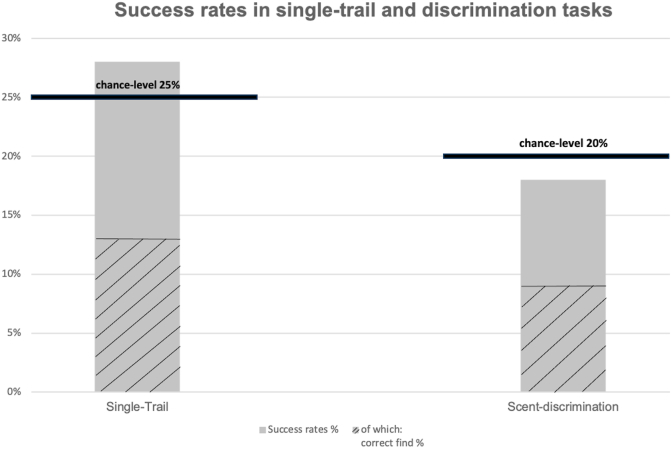
Success rates in the single-trail task and the scent-discrimination task relative to the expected chance level. Shaded areas indicate the proportion of trials resulting in a correct find within correctly classified trials. Horizontal lines denote the expected chance level for each task (25% for single-trail, 20% for scent discrimination).

The relationship between observed success rates and the respective chance expectations for both test formats is shown in Fig. 2.

### 4.3 Reproducibility

In the first round, all 70 dog–handler teams participated in the scent-discrimination task. Fourteen teams achieved a formally correct outcome (true positive or true negative). In addition, multiple inconclusive search outcomes were observed; six of these trials showed inconclusive search behaviour with a partial spatial approach toward the target trail, without a stable core running direction being identifiable. Overall performance in the first round was within the range expected by chance.

All 20 teams that had shown either a formally correct outcome or inconclusive search behaviour with partial approach toward the target trail were invited to a second round. As these results, despite individual correct decisions, remained within chance expectation, repeated testing served exclusively to assess reproducibility. In the second round, three teams achieved a true-positive outcome. One inconclusive search in spatial proximity to the target trail and one inconclusive response in a negative setup were statistically classified as false positives. Overall performance in the second round again remained within chance expectation.

As no performance above chance expectation was observed even among previously successful teams, five teams were invited to a third round to further examine reproducibility. In the third round, no team achieved a correct outcome. Four dogs followed incorrect trails, two of which led to incorrect target persons. One dog initiated two trails despite a negative setup and followed one of them over a considerable distance (exceeding 200 m); although the handler evaluated this behaviour as negative, the search was classified as inconclusive due to sustained trail commitment.

Each round was analysed separately and in its entirety against the corresponding chance expectation. Across all three rounds, success rates consistently remained within the range expected by chance, with no evidence of reproducible performance above chance level.

Repeated testing of teams that had previously achieved correct outcomes or shown partial indications of target-oriented behaviour showed no evidence of performance stabilisation. Success rates declined further in the second round and reached 0% in the third round, despite selective retesting based on these criteria.

### 4.4 Influencing factors

Exploratory analyses did not reveal systematic effects of environmental or training-related variables on performance. Across all examined factors, including temperature, relative humidity, start location, training duration, training frequency, certification status, and operational experience, success rates remained within the range expected by chance.

For example, in single-trail tests, correct initial orientation occurred in 28% of cases, closely matching the chance level of 25%. In scent-discrimination tasks, overall correct decisions were observed in 18% of trials, corresponding to the expected chance level of 20%.

Subgroup analyses across environmental conditions (e.g., temperature range: 2–32 °C; varying humidity levels) and training-related variables showed no consistent deviations from chance-level performance. Observed numerical differences between subgroups were small, inconsistent, and within the range of expected random variation (see Supplementary Table S1 and S2).

Repeated testing of selected teams did not demonstrate performance stabilisation. In the second round (n = 20), the success rate was 15% (3/20), remaining within the range expected by chance. In the third round (n = 5), no correct outcomes were observed (0/5; 0%). Despite targeted selection of teams that had previously achieved correct or directionally suggestive results, no reproducible above-chance performance was observed.

Performance remained within the range of chance expectation across all subgroups.

### 4.5 Overall outcome

Dogs frequently followed a single scent trail over extended distances but failed to reliably discriminate between multiple human scent trails under field conditions. Directional decisions and target identification remained at chance level, with no reproducible above-chance performance.

## 5 Discussion

Under double-blind, randomised field conditions, the mantrailing teams examined were not able to reliably select the target person's trail from competing alternatives. Performance levels in both the single-trail and scent discrimination tests remained within the range of chance expectation and showed no reproducibility across repeated test sessions. Thus, under strictly controlled conditions, no statistically significant evidence for reliable initial trail selection could be demonstrated.

These findings do not contradict the well-documented olfactory discrimination capabilities of dogs established in laboratory-based studies [[6], [7], [8]]. Rather, they highlight a pronounced discrepancy between highly controlled laboratory paradigms and the individual scent attribution required for forensically meaningful conclusions under realistic field conditions [[9], [10], [11], [12], [13]]. While laboratory studies largely minimise environmental variability, contextual information, and cognitive load, field searches are characterised by unstable, spatially diffuse, and context-dependent scent information, particularly at the critical decision point at the start of a search.

It must be taken into account that laboratory-based scent identification procedures and initial trail selection in mantrailing represent fundamentally different decision problems. Whereas scent identification line-ups require the assignment of a reference scent to a limited set of discrete, static comparison samples under highly controlled conditions [[6], [7], [8]], initial trail selection involves mapping an object-bound reference scent onto spatially distributed, dynamic environmental scent information within an open field context. This process is characterised by potentially fragmented, degraded, and context-dependent scent conditions and entails decision-making under increased uncertainty [[9], [10], [11], [12], [13],^19^]. Consequently, performance metrics derived from laboratory-based paradigms cannot be directly extrapolated to the forensically relevant task of initial trail selection.

A central observation of the present study was the differentiation between initial trail selection and subsequent trail following. Several dogs continued to follow a once-selected trail over considerable distances but failed at the initial selection of the correct starting trail. This pattern is consistent with a substantial influence of non-olfactory cues, particularly when olfactory information at the start point is weak or ambiguous. Expectation effects, cueing, and evaluation bias introduced by handlers are well documented in detection dog research and can influence both canine search behaviour and its interpretation [[14], [15], [16], [17], [18]].

Although no systematic effects of handler qualification or training experience were observed in the present data, variation in handler expertise and its potential influence on the interpretation of canine behaviour cannot be fully excluded and should be considered as a potential confounding factor.

A further consideration concerns the possibility that the lack of reproducibility may partly reflect methodological aspects of the study design. In particular, the selected performance proxy—initial directional decision—may be considered a stringent criterion that does not fully capture successful mantrailing as typically understood in operational contexts.

However, this measure was deliberately chosen, as initial trail selection represents the critical decision point for any subsequent tracking behaviour and is of primary relevance for forensic interpretation. Successful trail following after an incorrect initial decision does not constitute valid individual scent attribution.

It may also be argued that task difficulty was too high, particularly for non-professional teams. This interpretation appears unlikely. Trail age and trail length were deliberately kept at comparatively low levels (short trails of only a few hundred metres and relatively fresh scent conditions), and neither strict trail fidelity nor successful location of the target person was required. The task was therefore intentionally simplified and focused exclusively on the initial directional choice.

In this respect, the applied conditions were substantially less demanding than those typically required in formal certification tests, where longer and older trails are standard. At the same time, the initial choice of direction represents a fundamental component of operational mantrailing that is not explicitly assessed in many testing frameworks.

Moreover, repeated testing of selectively chosen teams did not result in improved performance, further arguing against task difficulty as the primary explanation.

Taken together, while methodological factors may have contributed to overall task demands, they are unlikely to account for the consistent absence of reproducible above-chance performance across all test conditions. Although trail age was standardised within a predefined window and trail length was deliberately kept low, some residual variation cannot be entirely excluded and should be considered when interpreting the results.

The occurrence of structured, map-consistent search trajectories along trails that had been planned but not used (“ghost trails”) further supports this interpretation. Such behavioural patterns cannot be sufficiently explained by purely olfactory control but correspond to known cueing and expectation effects as well as to the limitations of pseudo matching-to-sample–based decision strategies, as described in experimental and applied studies on the performance assessment of detection dogs [[13], [14], [15], [16], [17]]. Comparable phenomena are discussed in the literature as a methodological challenge in scent-based identification tasks [12,19].

From a learning-theoretical perspective, the results are consistent with mechanisms such as condition bias and overshadowing. Classical conditioning models demonstrate that predictable or consistently available cues can dominate decision-making processes, particularly when target-relevant signals are intermittent or uncertain [22,23]. Under operational and training conditions, contextual or social cues may therefore exert stronger control over behaviour than olfactory information [[14], [15], [16], [17], [18], [19]]. Increasing training experience may, under such conditions, stabilise search routines without improving the validity of individual scent attribution under cue-reduced, double-blind conditions.

Although no systematic effects of training experience, handler qualification, or related variables were observed in the present study, this does not necessarily contradict existing literature suggesting that increasing training experience can stabilise search routines. Rather, it indicates that under conditions of high uncertainty—particularly at the initial decision point where scent information may be fragmented, weak, or ambiguous—training may primarily reinforce general search strategies and cue utilisation rather than improve the validity of individual scent attribution.

From a learning-theoretical perspective, repeated exposure under such conditions may strengthen the use of stable, non-olfactory cues (e.g., contextual, spatial, or handler-related information) through processes such as cue competition, overshadowing, and condition bias. As a result, training experience may lead to more consistent behaviour without increasing accuracy relative to the target scent.

This interpretation is consistent with findings from detection dog research demonstrating that performance can be strongly influenced by expectancy effects, handler cues, and task structure, particularly when target-relevant olfactory information is limited or unreliable [[14], [15], [16], [17], [18]]. In this context, the absence of a measurable effect of training experience in the present study may reflect the dominance of non-olfactory control processes at the critical initial decision stage, rather than a lack of learning per se.

The statistical analysis underlines the robustness of these findings. Neither overall analyses nor subgroup analyses revealed performance levels exceeding chance expectation. Repeated testing of individual teams likewise failed to produce performance stabilisation, indicating that isolated correct decisions do not reflect a reproducible discrimination capability.

A methodological consideration concerns the timing and structure of testing and retesting. Retests were conducted on separate days, typically several weeks later, thereby reducing the likelihood that performance decline was attributable to acute fatigue effects.

In addition, the two test formats of the initial test round (single-trail and scent-discrimination tasks) were, as a rule, conducted on different days; only a small subset of teams completed both tasks on the same day under controlled conditions and with several hours between tests.

Importantly, search duration in the initial test round was standardised and limited to a maximum of 15 min per test across all teams. No indications of reduced performance were observed in teams completing both tasks on the same day.

While this design minimises fatigue-related confounding, retesting was implemented adaptively during the study and selection was based on prior performance characteristics. Although this approach allowed targeted assessment of reproducibility among apparently successful or borderline-performing teams, it may limit generalisability at the individual level. Notably, even under these conditions, no evidence of performance stabilisation was observed across repeated trials.

Discrepancies between the present results and studies reporting higher success rates can plausibly be explained by methodological differences. Study designs employing very short or very fresh trails, predefined starting directions, access to highly airborne scent information, or only single-blind procedures primarily assess trail following under favourable conditions and are more susceptible to cue-based strategies [[9], [10], [11], [12], [13]]. In contrast, the present study explicitly focused on initial individual scent-based decision-making under strict control of non-olfactory influences and was thus closely aligned with forensic standards and methodological recommendations [1,4,5].

## 6 Conclusions

Under double-blind, randomised field conditions, mantrailing dogs did not demonstrate a reproducible ability to select an individual target trail from competing alternatives based on a scent article. Directional decisions in both single-trail and scent-discrimination tasks remained within chance expectations and did not stabilise upon repeated testing.

These findings do not challenge the generally high olfactory discrimination capacity of dogs and are not transferable to detection dogs or scent-based search methods in general. Rather, they demonstrate a marked discrepancy between controlled laboratory conditions and the individual scent attribution required in mantrailing under realistic field conditions. The present data provide evidence that mantrailing, as practised within the tested operational and training context, achieves no robust and reproducible person-specific trail attribution at the critical decision point of initial trail selection.

Mantrailing appears particularly susceptible to non-olfactory influences due to its characteristic training structure and the interpretative role of handlers during the search process. Missing persons should therefore be searched for by other detection-based search methods, such as area search or non-individualised trailing, because these are less affected by these factors owing to clearly defined target stimuli and reduced handler-dependent decision components.

From a forensic perspective, mantrailing outcomes should therefore not be used as stand-alone evidence for person-specific attribution unless and until validity and reliability have been demonstrated under realistic, blinded field conditions. In particular, negative or incomplete searches should not be interpreted as providing directional or exclusionary information, given the demonstrated susceptibility of initial trail selection to cueing, condition bias, and contextual influences.

Future research should focus on mantrailing-specific validation approaches capable of disentangling olfactory from non-olfactory decision influences. This includes the development and systematic evaluation of training and testing paradigms that minimise cueing and condition bias, in order to determine whether reproducible above-chance performance is achievable under operational field conditions.

## Data availability statement

The datasets generated and analysed during the current study are not publicly available due to data protection and ethical restrictions but are available from the corresponding author upon reasonable request.

## Declaration of generative AI and AI-assisted technologies in the manuscript preparation process

During the preparation of this work, the author used ChatGPT (OpenAI) to assist with language editing, clarity, and structural refinement of the manuscript. The author critically reviewed and edited all generated content and takes full responsibility for the content of the published article.

## Funding

This research received no external funding. Open access publication is supported by the Faculty of Environment and Natural Sciences, 10.13039/501100022023Brandenburg University of Technology Cottbus–Senftenberg, Cottbus, Germany.

## Declaration of competing interest

The author declares no conflict of interest.
